# The Gnat and the Camel

**Published:** 1891-03-14

**Authors:** 


### The Doctor's Armchair.

### THE GNAT AND THE CAMEL.

One of the most striking figures of speech in all literature was used against certain learned persons not removed so much as two thousand years from our own times. "Ye blind guides," said a great teacher to those learned men, "ye blind guides, that strain out a gnat and swallow a camel." The picture of a number of grave and reputable men, endowed with the best culture of the times, carefully putting clean water through a filter lest they should inadvertently take in with the fluid a microscopic gnat; and then, when their filtering operations were finished, turning away, and, by a prodigious and boa-constrictor-like effort, gulping down a camel, was one which has never been surpassed for effectiveness and concentrated contempt. Learned men are often insusceptible to argument. Frequently they are vain, like pretty women; and, like pretty women, they must be approached in a p culiar way, or they will refuse to be approached at all. The pretty woman prefers men of distinction, and she loves to see flattery in the glance of their eyes and in their every movement. She insists upon hearing it in their speech. It would be considered brutal if one were to say that very generally such a woman is merely a nuisance. But a nuisance she is all the same, and it would be well if that were the end of the matter.

Like this woman, the learned man, for the most part, dearly loves flattery. If you will but take him at his own valuation he will pronounce you the best and most amiable fellow in the world. But hint a few contradictions, project a cloud or two of doubt across the sky of his serenity, whisper in the timidest of voices that Smith has made a discovery which may possibly put your learned friend into the second, or even the third place, and you are ruined in his estimation for ever.

This preface is suggested by the recent pilgrimage of London doctors to Berlin on the one hand, and the extraordinary apathy of the same doctors to a horrible cause of death that besieges this City of London every few weeks in the winter time, and which produces more suffering and does more harm to life and property in a single decade than consumption does in a century. We doctors who dwell in London have attained great proficiency in straining out gnats and swallowing camels. A learned German prematurely blazons before the world a new method of treating consumption, and hundreds of medical men from London rush to worship at his shrine, and to listen to his words as if they were the utterances of an oracle. Within a few weeks of the event, fog upon fog makes an Inferno of our city, chokes us, poisons us, crushes the life and spirit out of us, disables the strong, kills the less robust, hurries old men and women prematurely to their graves, and stops the breath of life in the infant and the child. How many doctors have a single word to say upon the subject? Dr. Richardson is the only man who has taken up his parable; and even he must feel by this time that he is nothing but "a voice crying in the wilderness."

Why should doctors charge themselves with the extinction of the deadly fog? asks a chorus of practitioners. Why? Why should a soldier fight when his country is invaded? Why should a parson preach and pray when vice is rampant? Why should the farmer sow his corn in the spring, and reap it in the autumn? Why do we eat when we are hungry, and put on flannel to protect ourselves from the winter's cold? To ask why the medical profession should charge itself with the duty of clearing London of its fogs is to show the brains of a tadpole, or, worse still, of a crocodile, that is said to weep especially hypocritical tears when it swallows a baby more than usually juicy and plump. Of course, it is the duty of the medical profession to open a crusade against the London fogs, and never to lay down its arms until the infidel is expelled from the noble city. Every rank and class of men has brains enough and manhood enough to possess a little patriotism, a little love of country and of city. Are we, doctors, the only persons to say that public injuries and calamities make no claim upon us? It is only doctors who know and understand the immeasurable damage done by London fogs. It is only they who see the enemy in his true proportions and ferocity, and only they therefore who can fight him with assured success. The power imposes the obligation.

"What can we do?" says the half-convinced man with a plea of pathetic helplessness in his tone! We can do nothing without a new motive power in our heads and hearts. But if we were once convinced that duty singled us out as the exterminators of the fog, and if we made up our minds as a body that we would obey the call of duty, we could bring such an amount of intelligent and urgent pressure to bear upon the Press, the public, and the authorities, as would drive away the filthy and poisonous enemy for ever in five years. Dr. Richardson tells us in two words how it is to be done, and there is no doubt he tells us truly. "Drain the surface thoroughly," he says in effect, "and then you will have no vapours to rise. Put an end to the coal fires in your houses and factories, and then you will have no smoke to fall." The fog consists entirely of vapour and smoke. If both are prevented from being formed, fogs will be impossible. That they can be prevented is as certain as that drains can be laid and gas manufactured.

The man of feeble and timid mind whimpers about the cost. He is the sort of fellow that cries when his own finger is pricked with a pin, but sees his brother or his cousin gored by a mad bull without emotion. He will dismiss his cook if she asks him twenty-five pounds in wages instead of twenty-four, but will invest twenty thousand pounds in a bubble company without a single moment's thought of the future. Of course it would cost money to expel the fog fiend once and for ever by Dr. Richardson's methods; money in rates, and money in increased private expenditure for a time. But such money would be merely invested, and it would yield a large return of interest even in money; and not in money only, but in health, in cheerfulness, in efficiency for work, in long life, in diminished expenses of all sorts; and, in short, in everything that makes life worth living. *The Daily Graphic* has taken up the subject under the inspiration of a letter from Mr. James Knowles, of the *Nineteenth Century*. As yet the interest displayed by the public is of a very languid character. That is, no doubt, because the public feels that there is no hope of anything being done. The thing has been talked about so often, and with no result, that the practical English mind puts its hands into its pockets, and shrugs its shoulders—if we may be permitted for a moment to imagine that a mind has hands and pockets and shoulders—and simply declines to think anything more at all about the matter.

The *non possumus* position, we venture to say, is not only wrong-headed but impossible. London, within an easily measurable distance of time, will become uninhabitable if things are left to take their present course. That, no doubt, will be considered a somewhat impotent argument by those persons who are so brutally selfish as to care not a single straw for anything beyond the urgent necessities of the present. But from a doctor's point of view London during a considerable portion of the year is quite uninhabitable now. Doctors know this to their cost, and more especially the consulting physicians and the higher class of general practitioners. During a severe and protracted fog nobody comes to London to consult a physician who can possibly help it, and no family continues to live in London who can afford to live elsewhere. It is clear, therefore, that both self interest and patriotism combine to place upon the doctors' shoulders the responsibility of ridding London of its fogs. We shall watch with much interest to see what Dr. Richardson and his fellow scientists will attempt between now and next November.

